# Editorial: Estrogens and neurodegeneration: a link between menopause and Alzheimer’s disease in women

**DOI:** 10.3389/fmolb.2025.1727385

**Published:** 2025-11-07

**Authors:** Manuela Leri, Andrea Bertolini, Mario Diaz, Roberta Marongiu

**Affiliations:** 1 Department of Biomedical, Experimental, and Clinical Sciences “Mario Serio” University of Florence, Florence, Italy; 2 Department of Surgical, Medical, Molecular and Critical Area Pathology, University of Pisa, Pisa, Italy; 3 Universitary Institute of Neurosciences (IUNE), University of La Laguna, San Cristóbal de La Laguna, Spain; 4 Department of Neurological Surgery, Weill Cornell Medicine, New York, NY, United States; 5 Department of Genetic Medicine, Weill Cornell Medicine, New York, NY, United States; 6 Feil Family Brain and Mind Institute, Weill Cornell Medicine, New York, NY, United States

**Keywords:** estrogen, menopause, Alzheimer’s disease, sex differences, neurodegeneration

Alzheimer’s disease (AD) remains one of the most pressing public health challenges of our time. One of its most striking, yet historically underexplored, features is its disproportionate impact on women (Alzheimer’s Association Report, 2024). Nearly two-thirds of all AD cases occur in women. While women do live longer on average, longevity alone does not explain this gap. Increasing evidence support that the biological changes during the menopause transition represents a neurological turning point that may accelerate the trajectory of brain aging in women. Understanding why women are disproportionately affected by AD has therefore become a critical challenge in neuroscience and aging research.

This Research Topic explores menopause, and the hormonal upheaval that accompanies it, as a critical inflection point in neurodegeneration and highlights emerging mechanisms and therapeutic strategies that reflect the complexity of the female brain across the lifespan. For decades, estrogen has been recognized as a key neuroprotective hormone. It maintains synaptic integrity, supports mitochondrial function, regulates cerebral blood flow, and modulates neuroinflammation (Galea et al., 2017). However, the field has often viewed menopause through the narrow lens of “estrogen loss,” as if it were a single hormonal event. In reality, menopause triggers a cascade of hormonal shifts–including endocrine, metabolic, vascular, and neurological changes - and only now are we beginning to understand how these changes might interact to influence neurodegenerative processes. The articles in this Research Topic move the field beyond estrogen alone and toward a more integrated understanding of endocrine aging in women.

Mervosh and Devi revisit the role of hormonal changes at menopause with renewed depth. They emphasize both classical genomic estrogen receptor signalling and rapid, non-genomic actions that influence cognitive resilience. Importantly, they situate estrogen within a network of interacting risk factors—including APOE genotype and cardiovascular health—highlighting why hormone therapy (HT) has shown heterogeneous results across studies. Rather than asking “Does HT work?”, their review reframes the conversation: When, how, and for whom does HT provide benefit? This perspective reinforces the “critical window hypothesis,” which proposes that HT is most effective when initiated near the onset of menopause, before extensive neural aging or pathology occur (Maki, 2013).

However, estrogen is only one part of the overall picture. Xue et al. draw attention to follicle-stimulating hormone (FSH), which rises dramatically during menopause, and may itself have direct pathological effects on the brain (Xue et al.). Their review links elevated FSH levels to key AD-related pathological mechanisms, including neuroinflammation, metabolic dysfunction, vascular changes, and amyloid pathology. By proposing FSH as an active driver of neurodegeneration and a potential therapeutic target, they expand the field’s view of menopause from simple estrogen deficiency to the broader endocrine shifts that characterize midlife in women.

Building on this expanded endocrine perspective, Lizcano et al. introduce an innovative therapeutic concept: combining estrogens with GLP-1 receptor agonists (Lizcano et al.). Traditionally used in metabolic disorders like type 2 diabetes, GLP-1 agonists are now emerging as neuroprotective agents for neurodegenerative disorders like AD and Parkinson’s disease (Hölscher, 2024). The authors argue that combined use of GLP-1 agonists with estrogenic compounds may offer a dual-action benefit, simultaneously targeting mitochondrial dysfunction, oxidative stress, and insulin signalling deficits. This synergy is particularly relevant in postmenopausal women, who often experience simultaneous endocrine and metabolic shifts that contribute to neurodegenerative risk.

Complementing these conceptual advances are a series of empirical studies that explore how hormonal transitions manifest in biological systems. Watts et al., using data from the Investigating Gains in Neurocognition in an Intervention Trial of Exercise (IGNITE) cohort (Erickson et al., 2019), investigate how cumulative lifetime exposure to estrogen, from natural cycles, oral contraceptives, or hormone therapy, relates to cognitive performance in later life (Watts et al.). Their findings lend support to the idea that the timing of hormonal exposure matter greatly. Although oophorectomy prior to natural menopause was not associated with poorer cognition later in life, women who initiated HT near the time of oophorectomy showed better memory and visuospatial performance outcomes. These results reinforce insights from clinical trials that timing is not a trivial variable, but a potential determinant of therapeutic efficacy.

Complementing the human data, work by Marongiu et al. provide critical preclinical evidence that brain vulnerability begins even before menopause is complete. Using a unique perimenopausal mouse model that mimics the irregular hormone fluctuations of the menopause transition, they demonstrate that even partial ovarian hormone decline can lead to increased amyloid deposition and glial activation in the hippocampus (Marongiu et al.). These neuropathological changes appeared before any cognitive symptoms emerged, suggesting that brain vulnerability begins earlier than previously assumed. This work delivers two major insights: first, that the earliest window of risk occurs during the menopause transition, not only after menopause; and second, that prevention strategies may need to begin before menopause is complete. Collectively, this Research Topic does not present a single linear explanation, but rather a coherent, multidimensional framework for understanding why women are disproportionately affected by AD. Several unifying themes emerge: 1) Mechanisms are multilayered, as menopause disrupts synaptic plasticity, mitochondrial energy production, vascular function, and immune regulation simultaneously, creating a convergence of vulnerabilities relevant to AD; 2) Human and animal data reinforce the concept that both timing for HT and context matter (e.g., age, genetic background); 3) Other hormones, especially FSH, may play active roles; 4) Translational potential for early HT intervention and combination therapies is high, but clinical heterogeneity must be addressed. Inconsistent clinical trial outcomes highlight the need to account for heterogeneity in hormone formulation, dose, route, timing, genetic predisposition, and surgical vs. natural menopause into precision treatment models.

Menopause is not merely a reproductive milestone; it is a neurobiological turning point that reshapes brain vulnerability. The articles in this Research Topic shift the field from simplistic models of estrogen decline to a sophisticated understanding of menopause as a multi-hormonal, metabolic, immune, and vascular inflection point in women’s brain aging. If there is a unifying message in these diverse contributions, it is that timing, personalization, and integration must guide the future of preventions and treatment. Hormone-related interventions cannot be considered in isolation from metabolic health, nor can cognitive decline be fully understood without accounting for lifetime hormonal exposure. The path forward lies in developing interventions that are not only sex-specific but biologically tailored, accounting for hormonal profiles, genetic predispositions, and the precise timing of menopausal transitions.

This Research Topic takes us closer to a future in which women’s brain health is understood on its own biologically terms. By expanding our understanding of how neurodegeneration unfolds in the female brain, it paves the way for earlier, more effective, and personalized strategies to delay or prevent AD–strategies that truly reflect the complexity of women’s biology across the lifespan.
